# High iron requirement for growth, photosynthesis, and low-light acclimation in the coastal cyanobacterium *Synechococcus bacillaris*

**DOI:** 10.3389/fmicb.2015.00561

**Published:** 2015-06-18

**Authors:** William G. Sunda, Susan A. Huntsman

**Affiliations:** Beaufort Laboratory, National Ocean Service, National Oceanic and Atmospheric Administration, BeaufortNC, USA

**Keywords:** iron growth limitation, cyanobacteria, evolution, low light acclimation, iron-light co-limitation, deep chlorophyll maximum, cellular Fe:C ratio, photosynthesis

## Abstract

Iron limits carbon fixation in much of the modern ocean due to the very low solubility of ferric iron in oxygenated ocean waters. We examined iron-limitation of growth rate under varying light intensities in the coastal cyanobacterium *Synechococcus bacillaris*, a descendent of the oxygenic phototrophs that evolved ca. 3 billion years ago when the ocean was reducing and iron was present at much higher concentrations as soluble Fe(II). Decreasing light intensity increased the cellular iron:carbon (Fe:C) ratio needed to support a given growth rate, indicating that iron and light may co-limit the growth of *Synechococcus* in the ocean, as shown previously for eukaryotic phytoplankton. The cellular Fe:C ratios needed to support a given growth rate were 5- to 8-fold higher than ratios for coastal eukaryotic algae growing under the same light conditions. The higher iron requirements for growth in the coastal cyanobacterium may be largely caused by the high demand for iron in photosynthesis, and to higher ratios of iron-rich photosystem I to iron-poor photosystem II in *Synechococcus* than in eukaryotic algae. This high iron requirement may also be vestigial and represent an adaptation to the much higher iron levels in the ancient reducing ocean. Due to the high cellular iron requirement for photosynthesis and growth, and for low light acclimation, *Synechococcus* may be excluded from many low-iron and low-light environments. Indeed, it decreases rapidly with depth within the ocean’s deep chlorophyll maximum (DCM) where iron and light levels are low, and lower-iron requiring picoeukaryotes typically dominate the biomass of phytoplankton community within the mid to lower DCM.

## Introduction

Phytoplankton represent the base of the marine food web and are major drivers of the biogeochemical cycling of carbon (C) and nitrogen (N), and the ability of the ocean to sequester carbon dioxide (CO_2_) via the biological carbon pump (Martin, 1990; Sigman and Boyle, 2000). Hence, they are important in regulating atmospheric CO_2_ and related greenhouse warming of the planet. Due to its extremely low concentration, iron (Fe) limits phytoplankton productivity in high nitrate-low chlorophyll (Chl) ocean waters which occupy ∼30% of the world ocean (Moore et al., 2013). It also limits dinitrogen (N_2_) fixation in large regions of the thermally stratified tropical and subtropical ocean, due to the high metabolic requirement for iron in this process (Sohm et al., 2011).

Iron may also limit phytoplankton growth in the deep chlorophyll maximum (DCM) of thermally stratified regions of the ocean, such as the subtropical mid-ocean gyres (Sunda and Huntsman, 1997; Sedwick et al., 2005; Hopkinson and Barbeau, 2008). These chlorophyll maxima are persistent features at the bottom of the euphotic zone of virtually all stratified oceanic regions and result both from increased Chl:C ratios in the phytoplankton community in response to light limitation and from increased productivity linked to the diffusive and advective flux of limiting nutrients (nitrate and iron) from deeper aphotic depths (Cullen, 1982; Huisman et al., 2006; Hopkinson and Barbeau, 2008; Ledwell et al., 2008). Phytoplankton numbers in the upper euphotic zones of these stratified oceanic regions are often dominated by picocyanobacteria *Synechococcus* and *Prochlorococcus*, which have cell diameters of 0.5–1.5 μm and account for an estimated 25% of marine primary production (Waterbury et al., 1979; Flombaum et al., 2013). *Synechococcus* is widely distributed in both coastal and oceanic waters while *Prochlorococcus* is restricted to nutrient limited oceanic waters and is often numerically dominant in these environments (Olson et al., 1990; Li, 1995; Bibby et al., 2009; Flombaum et al., 2013).

Iron is essential to the metabolism and growth of all organisms and is especially important in phytoplankton because of its presence in iron–sulfur and cytochrome proteins involved in photosynthetic electron transport (Raven, 1990). Cellular models and empirical measurements indicate that ≥50% of the metabolic iron in eukaryotic phytoplankton (e.g., diatoms) occurs in the photosynthetic apparatus (PA; Raven, 1990; Strzepek and Harrison, 2004). Because cells up-regulate the capacity of the PA during subsaturating light conditions, iron growth requirements increase under reduced irradiance, which can lead to iron-light co-limitation of phytoplankton growth (Sunda and Huntsman, 1997, 2011). Relationships between intracellular iron concentrations and growth rate under varying light intensities have been documented in eukaryotic phytoplankton (Sunda and Huntsman, 1997, 2011; Strzepek and Harrison, 2004), but such relationships have not been reported in picocyanobacteria, despite their abundance and contribution to primary production in the ocean.

In addition to their ecological importance, cyanobacteria such as *Synechococcus* are also of evolutionary interest because they are direct descendants of the earliest oxygenic phototrophs, which evolved ∼3 billion years ago when the chemistry of the ocean was far different than it is today (Blankenship et al., 2007). At that time the ocean was reducing and contained no free oxygen, and iron was present as soluble iron(II) at orders of magnitude higher concentrations than occur in the modern ocean (Osterberg, 1974; da Silva and Williams, 1991; Saito et al., 2003). Cyanobacteria have been proposed to possess a much higher cellular growth requirement for iron than more recently evolved eukaryotic algae, as a vestige of the high availability of iron in the ancient ocean (Brand, 1991; Saito et al., 2003). Brand’s (1991) hypothesis was based on the much higher subsistence Fe:P requirement in six coastal and oceanic *Synechococcus* strains (2–38 μmol mol^-1^) whose growth had been reduced to zero by iron-limitation, relative to subsistence Fe:P values for eukaryotic algae: <0.1–0.4 for oceanic species and 0.8–10 for coastal species. These subsistence Fe:P values for *Synechococcus* translate to Fe:C ratios of 15–290 μmol mol^-1^ based on the measured C:P molar ratio in this genus of 132 ± 21 (Bertilsson et al., 2003). Subsequent experiments gave similar Fe:C ranges for iron-limited *Synechococcus* strains: 42–150 μmol mol^-1^ for coastal strains and 27–117 for oceanic strains (Wilhelm et al., 1996; Kudo and Harrison, 1997; Quesnel, 2009). However, none to these studies presented detailed relationships between specific growth rates and cellular Fe:C ratios.

In the present experiments we measured relationships among concentrations of biologically available dissolved inorganic ferric iron species (Fe′), cellular iron uptake rate, cellular Fe:C ratio, specific growth rate, and chlorophyll *a* (Chl *a*) in the marine picocyanobacterium *Synechococcus bacillaris* (CCMP 1333), isolated from coastal waters where extant iron concentrations are much higher than in the open ocean (Sunda and Huntsman, 1995). These relationships were measured at saturating light (500 μmol quanta m^-2^ s^-1^), and two lower growth-limiting light intensities (160 and 50 μmol quanta m^-2^ s^-1^). In addition, the high light experiments were measured at two environmentally relevant free cupric ion concentrations (0.16 and 16 pM) to determine if copper influenced iron uptake and growth limitation as had been found to occur with eukaryotic phytoplankton due to its requirement in a high affinity cellular iron uptake system (Maldonado et al., 2006). The experiments had three major objectives: (1) to determine if iron requirements for growth in *Synechococcus* increase under light limitation as predicted from theory (Raven, 1990) and previously shown in coastal eukaryotic algal species (Sunda and Huntsman, 1997, 2011), (2) to compare the cellular iron requirements for growth at varying light intensities in this coastal cyanobacterium with those determined previously in eukaryotic algal species isolated from similar high iron coastal environments, and (3) to determine if variations in cupric ion concentration influence iron uptake or iron-limitation of growth rate. *S. bacillaris* (CCMP 1333) was also chosen for these experiments because previous experiments with this strain had shown that it had unusual metal requirements and sensitivities consistent with a vestigial adaptation to the trace metal conditions in the primordial anoxic ocean where concentrations of iron, manganese, and cobalt were projected to be much higher than in the present day ocean, while concentrations of zinc, copper, and cadmium are believed to have been much lower (Osterberg, 1974; da Silva and Williams, 1991; Saito et al., 2003). *S. bacillaris* (CCMP 1333) was found to have an absolute requirement for cobalt and none for zinc, in contrast to the situation with more recently evolved eukaryotic algal species (Sunda and Huntsman, 1995), while it had a much greater sensitivity to copper and cadmium toxicity than marine eukaryotic algae (Brand et al., 1986). Both of these attributes (a need for cobalt but not zinc and high sensitivity to copper and cadmium toxicity) are consistent with adaptations to the trace metal conditions in the primordial anoxic ocean (Brand et al., 1986; Saito et al., 2003). So we wanted to find out if this *S. bacillaris* strain also had an unusually high growth requirement for iron, consistent with an adaptation to the high iron concentrations in the early reducing ocean. The subsistence Fe:P ratios have been previously measured in this isolate (and found to be high; Brand, 1991), but the iron requirements for growth (in terms of intracellular Fe:C ratios) have not yet been determined. In fact there has never been a comparison of the growth requirements of marine *Synechococcus* strains and eukaryotic algal species isolated from similar environments and examined under the same culture conditions of temperature, photoperiod, and light source and intensity, all of which influence iron growth requirements in eukaryotic algae (Sunda and Huntsman, 2011) and likely cyanobacteria as well.

## Materials and Methods

Iron uptake and growth experiments were conducted at 20°C with acclimated cultures of *S. bacillaris* (CCMP 1333; formerly Syn) using previously employed methods (Sunda and Huntsman, 1995, 2011). The experiments determined relationships among controlling variables (light intensity and the concentration of dissolved ferric iron hydrolysis species, Fe′) and relevant dependent variables (cellular Fe:C ratios, cellular iron uptake rates, Chl *a*, and specific growth rate). The experiments were conducted under a 14:10 h light:dark cycle with light provided by Vita-Lite fluorescent bulbs. Two separate experiments were run. The first was conducted at a high, growth-saturating light intensity (500 μmol quanta m^-2^ s^-1^) and two non-toxic free cupric ion concentrations (0.16 and 16 pM). A second experiment was conducted at the lower cupric ion concentration (0.16 pM) and light intensities reduced to 160 and 50 μmol quanta m^-2^ s^-1^ using neutral density screens (Sunda and Huntsman, 1997). Due to the difficulty in directly measuring the concentrations and volumes of the small *S. bacillaris* cells, biomass was determined from ^14^C measurements of cell carbon.

An axenic culture of *S. bacillaris* (CCMP 1333; formerly Syn) was obtained from the Provasoli-Guillard National Center for Marine Algae, East Boothbay, Maine and maintained in f/8 medium (Guillard and Ryther, 1962) using sterile technique. Cyanobacterial cells were grown in 450-mL polycarbonate bottles containing 200–250 mL of 36 salinity seawater medium. The experiments were conducted in Gulf Stream seawater that had been stored in the dark and cold (4°C) for several months and pre-filtered through 0.4 μm pore Nuclepore filters to remove naturally occurring phytoplankton. The filtered seawater was enriched with 32 μM NaNO_3_, 2 μM Na_2_HPO_4_, 40 μM Na_2_SiO_3_, 10 nM Na_2_SeO_3_, 0.074 nM vitamin B_12_, 0.4 nM biotin, and 60 nM thiamin. Trace metal ion buffer systems containing the chelator ethylenediaminetetraacetate (EDTA) were added to quantify and control free trace metal ion concentrations. These buffers consisted of 0.1 mM EDTA, and various concentrations of trace metals: 5.6 μM Mn, 31 μM Zn, 40 nM Co, 100 nM Ni, 0.13 or 13 μM Cu, and varied concentrations Fe. The higher Cu was added as CuEDTA to insure that the “free” EDTA concentration did not change with the Cu addition (Sunda et al., 2005). Free ion concentrations of Zn, Co, Mn, Ni, and Cu, and dissolved inorganic concentrations of iron were computed from the total metal concentration and the extent of metal complexation by EDTA and inorganic ligands (Sunda et al., 2005). The computed molar free ion concentrations of zinc, cobalt, manganese, and nickel were 5 nM, 15 pM, 500 nM, and 0.2 pM, respectively. The free cupric ion concentration was 0.16 pM at the lower Cu concentration (0.13 μM Cu) and 16 pM at the higher Cu level (13 μM).

Uptake of iron by phytoplankton in EDTA-buffered seawater media is related to [Fe′], the concentration of dissolved inorganic ferric hydrolysis species [Fe(OH)_2_^+^, Fe(OH)_3_, and Fe(OH)_4_^-^; Shaked et al., 2005]. Due to photochemical redox cycling, [Fe′] is dependent on light, and is higher during the light period than in the dark (Sunda and Huntsman, 2003, 2011). [Fe′] during the light and the dark periods were computed from iron-EDTA chelation data measured as a function of light intensity and pH at 20°C using the same fluorescent lighting as used in the culture experiments (Sunda and Huntsman, 2003). These data were used to compute the mean [Fe′] over the daily light:dark cycle (Sunda et al., 2005).

Prior to culture experiments, the cells were grown in ^14^C-labeled media at the experimental light intensity for 5–7 cell generations in an iron-limiting medium containing 30 nM iron ([Fe′] = 50–90 pM, depending on the light intensity) and low cupric ion concentration (0.16 pM). They were then inoculated into a fresh set of experimental media containing the same ^14^C-bicarbonate label and a range of iron concentrations radiolabeled with ^59^Fe [both nuclides obtained from Amersham (now GE Healthcare)]. The cells were inoculated at biomass levels of 0.3–0.7 μmol cell carbon per liter of culture, with the higher biomass inocula used for very low Fe′ levels, where less algal growth was expected. They were grown for 5–7 cell generations, except at very low Fe′ concentrations and low final specific growth rates (<0.1 d^-1^), where the cells grew for only 3–4 generations before their growth rates declined due to iron starvation. Total cell carbon per liter of culture was determined daily in the middle of the light period by filtering the cells through 0.4 μm-pore Nuclepore filters and measuring their total cell carbon from liquid scintillation counts of cellular ^14^C (Welschmeyer and Lorenzen, 1984; Sunda and Huntsman, 1995). Specific growth rate was determined by linear regression of ln cell carbon (per liter culture) vs. time during the exponential phase of growth. These regressions were highly significant with mean R^2^ values of 0.9968 ± 0.0035 for treatments with growth rates ≥0.11 d^-1^.

Cellular iron was measured in exponentially growing cultures in the middle of the light period at the same time as other cell measurements. Cells were filtered onto 0.4 μm-pore Nuclepore filters, and the filtrate was retained for measurement of blank values, which consisted of a fresh set of filters through which the original filtrates were passed. Both sets of filters were immediately rinsed with filtered seawater and then exposed for 2 min to Ti-EDTA-citrate (in seawater) to reductively remove iron oxides and iron bound to cell or filter surfaces (Hudson and Morel, 1989). The moles of intracellular Fe per liter of medium were determined in the Ti-washed cells by gamma counting of ^59^Fe on the culture and blank filters and in unfiltered aliquots of the cultures (Sunda and Huntsman, 1995). Subsequently, the filtered cells were measured for organic C by liquid scintillation counts of particulate ^14^C (Welschmeyer and Lorenzen, 1984; Sunda and Huntsman, 1995). Cellular iron is reported as Fe:C ratios. Average daily iron uptake rates were computed by multiplying the cellular Fe:C ratio by the specific growth rate (Sunda and Huntsman, 1995).

Chl *a* and culture pH were also measured. For the former, the cells were filtered onto 0.4 μm pore polycarbonate filters, extracted into a mixture of 45% (v/v) dimethylsulfoxide, 45% acetone, and 10% water and fluorometrically measured for Chl *a* (Sunda and Huntsman, 1995). Chl *a* was normalized to cell C to yield cell Chl *a*:C molar ratios. Culture pH was measured with a glass electrode against NBS pH standards (Sunda and Huntsman, 2003). The mean pH at the time of sampling for cell Fe:C and Chl:C ratios was 8.2 ± 0.1 (±SD).

## Results

**FIGURE 1:**
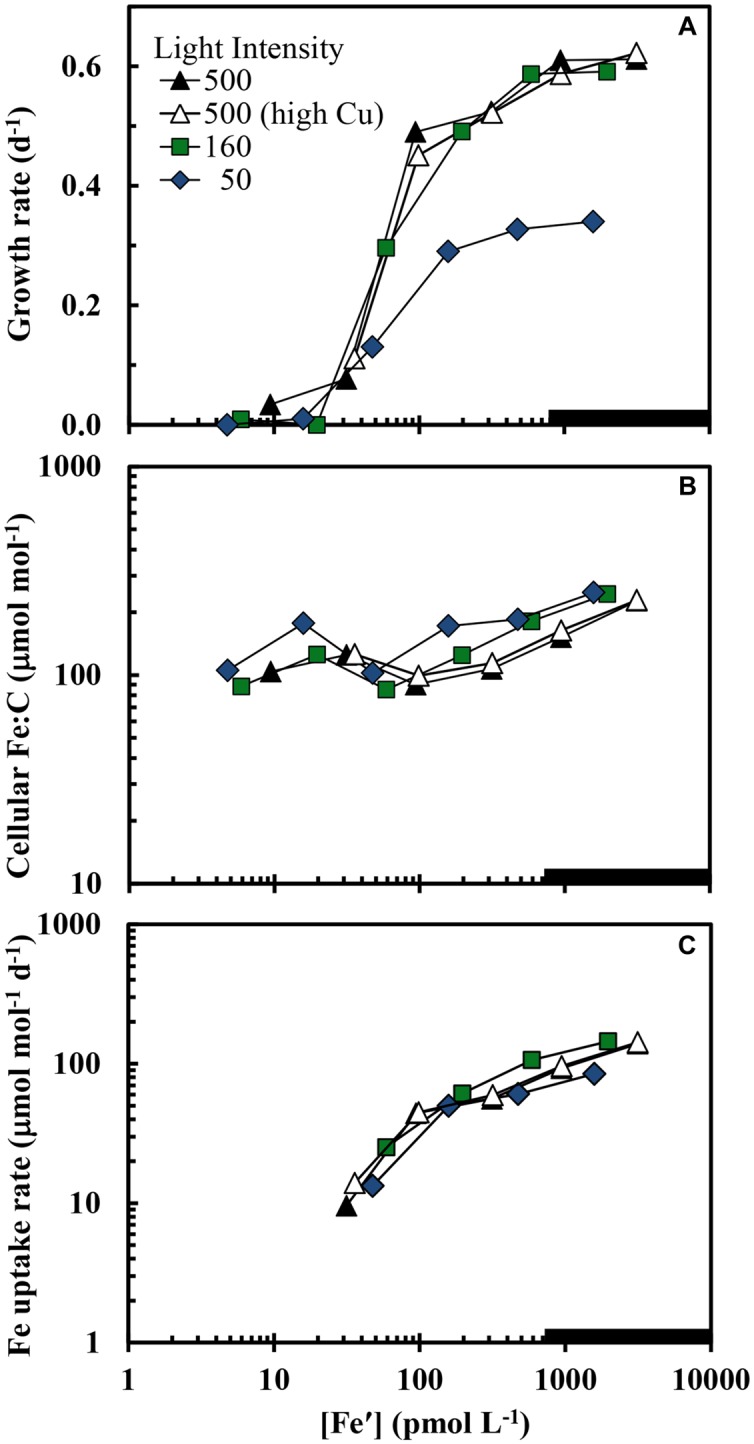
**(A) Specific growth rate, **(B)** cellular Fe:C ratio, and **(C)** carbon normalized cellular iron uptake rate (μmol [mol C]^-1^ d^-1^) in *Synechococcus bacillaris* plotted as functions of the average daily concentration of dissolved inorganic ferric hydrolysis species ([Fe′]) in picomolar (pM) units.** There were four separate treatments: (1) high light intensity (500 μmol quanta m^-2^ s^-1^) and low cupric ion concentration ([Cu^2+^] = 0.16 pM; solid black triangles), (2) high light intensity (500 μmol quanta m^-2^ s^-1^) and a higher cupric ion concentration ([Cu^2+^] = 16 pM; open triangles), (3) intermediate light intensity (160 μmol quanta m^-2^ s^-1^) and low copper ([Cu^2+^] = 0.16 pM; green squares), and (4) low light intensity (50 μmol quanta m^-2^ s^-1^) and low copper ([Cu^2+^] = 0.16 pM; blue diamonds). The thick solid line on the x-axis shows the region where iron hydroxides precipitate (Sunda and Huntsman, 1995). Within this region [Fe′] should no longer continue to increase and the x-axis values indicate proportional increases the total iron concentration.

**FIGURE 2:**
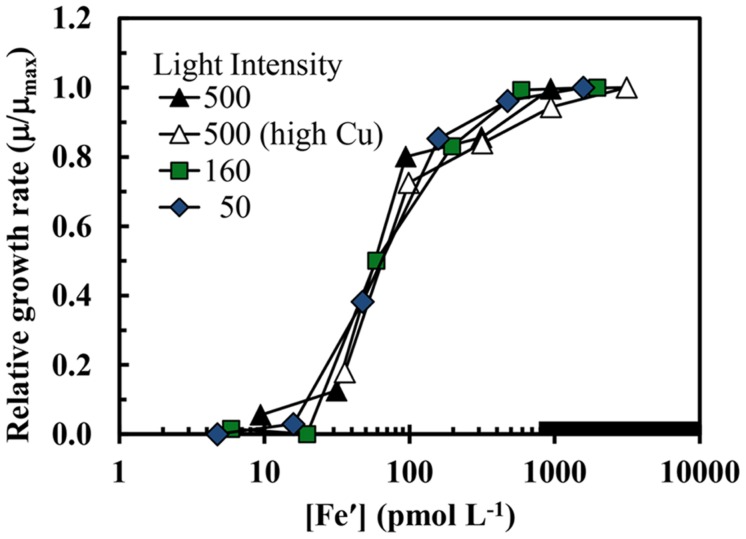
**The iron-limited growth rate normalized to the maximum iron sufficient rate (μ/μ_max_) in *S. bacillaris* plotted as a function of Fe′ concentration.** The treatments and symbols are defined in brief on the figure and in detail in the legend of **Figure 1**. The low copper data (all but the open triangles) were previously published in Sunda and Huntsman (1997).

**FIGURE 3:**
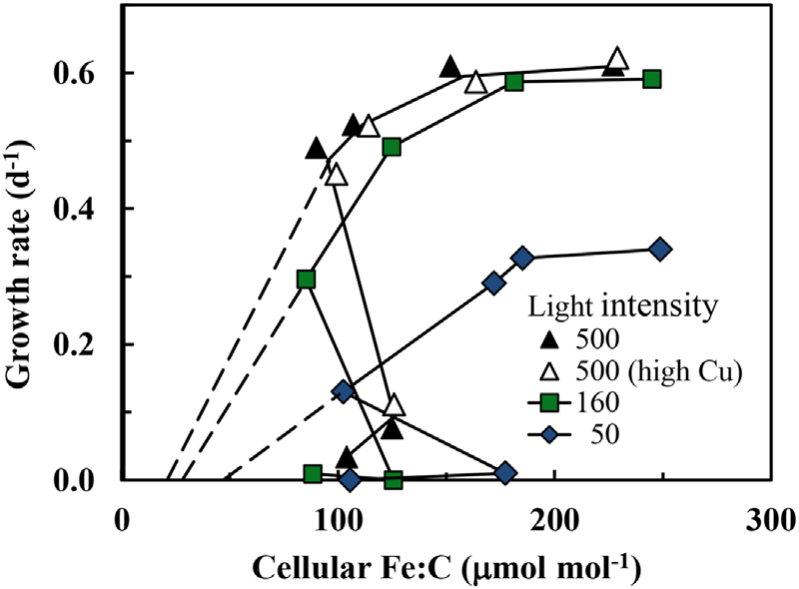
**Relationships between specific growth rate and cellular Fe:C molar ratio in *S. bacillaris* at different light intensities.** The treatments and symbols are defined in brief on the figure and in detail in the legend of **Figure 1**. The dashed lines give the expected relationships between the specific growth rate and metabolic iron normalized to cell C at the three light intensities based on the relationships between specific growth rate and cellular Fe:C in coastal eukaryotic species *Thalassiosira pseudonana* and *Prorocentrum minimum* (Sunda and Huntsman, 1997, 2011).

**FIGURE 4:**
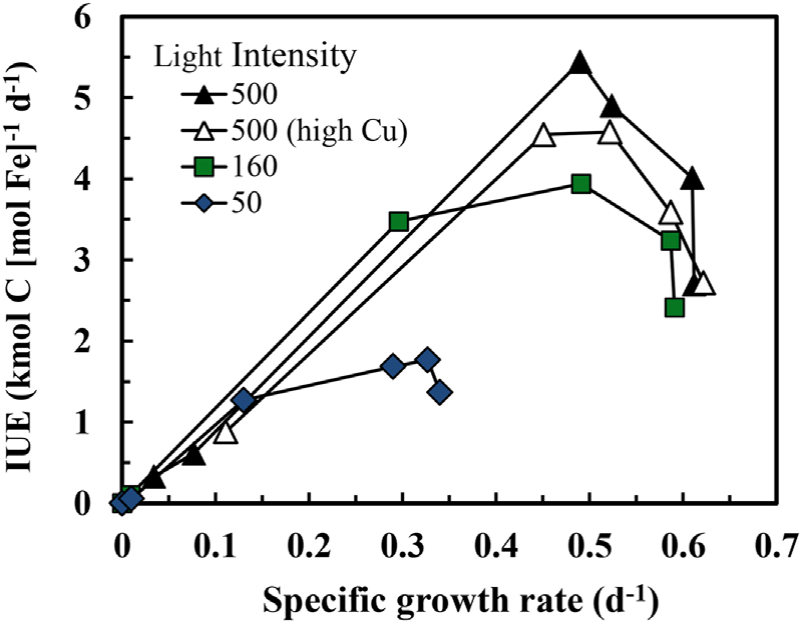
**Relationship between iron use efficiency (IUE) and specific growth rate in iron-limited and iron-sufficient cultures at three light intensities over a range of iron concentrations.** The treatments and symbols are defined in brief on the figure and in detail in the legend of **Figure 1**.

**FIGURE 5:**
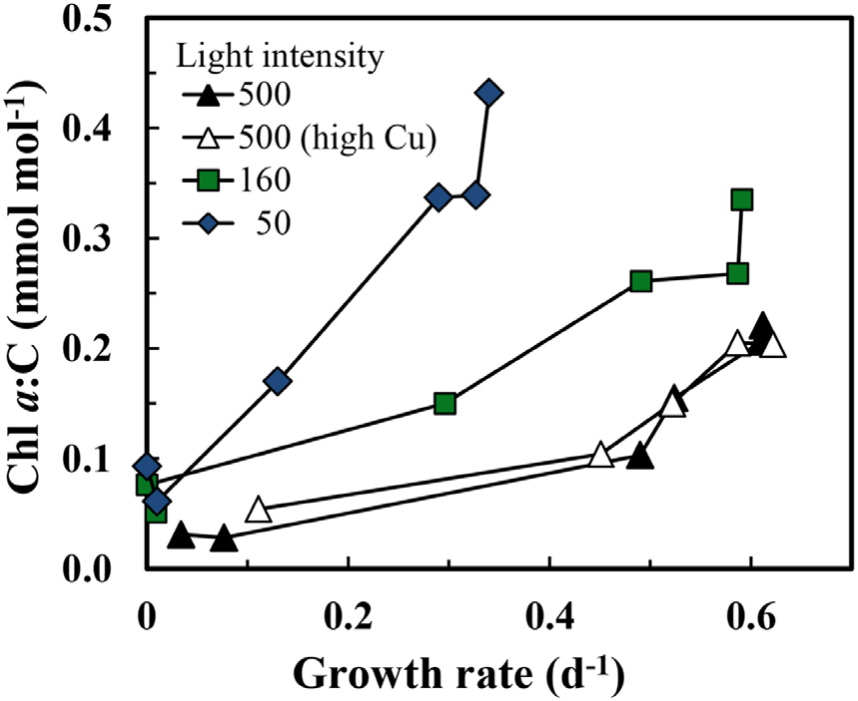
**Relationships between cellular Chl *a*:C ratio and specific growth rate in iron-limited and iron-sufficient cells grown at the three light intensities: 500, 160, and 50 μmol quanta m^-2^ s^-1^.** The treatments and symbols are defined in brief on the figure and in detail in the legend of **Figure 1**.

The maximum iron-sufficient specific growth rate of *S. bacillaris* was 0.62 d^-1^ at the highest light intensity (500 μmol quanta m^-2^ s^-1^), and decreased by 4 and 48%, respectively, at intensities of 160 and 50 μmol photons m^-2^ s^-1^ (**Figure 1A**). In the first experiment at the highest light intensity, the results obtained at the two free cupric ion concentrations (0.16 and 16 pM) were similar to one another with little difference between the two treatments for all experimental cellular parameters (**Figures 1–5**). The specific growth rate at the higher cupric ion concentration minus that at the lower averaged -0.004 ± 0.0285 for all five iron treatments, while the Fe:C values and iron uptake rates at the higher Cu ion concentration were slightly higher (5.2 ± 5.6 and 3.5 ± 2.0% higher, respectively) than those at the lower copper level. The relationship between the iron-limited specific growth rate normalized to the maximum iron-sufficient rate (μ/μ_max_) and Fe′ concentration ([Fe′]) also was similar at all light intensities and copper levels (**Figure 2**). Iron-limited the growth rate of *S. bacillaris* at an [Fe′] below ca. 700 pM, just below the maximum solubility limit for precipitation of ferric hydroxides (measured empirically in a previous experiment at the same pH, salinity, temperature, and photoperiod; Sunda and Huntsman, 1995; **Figures 1A** and **2**). Growth rates decreased to near zero at [Fe′] ≤20 pM.

Computed cellular iron uptake rates increased by 15-fold (from 10 to 145 μmol Fe [mol C]^-1^ d^-1^) over the range of specific growth rates (≥0.13 d^-1^) and [Fe′] (≥30 pM) where these rates could be determined (**Figure 1C**). Below 0.13 d^-1^ the growth rate of the cells was insufficient to establish a steady state relationship among the cellular Fe:C, cellular iron uptake rate and specific growth rate (see Eq. 1 below). Cellular Fe:C ratios increased by a lesser amount (3-fold; from 85 to 250 μmol Fe [mol C^-1^]) over the same [Fe′] range (**Figure 1B**). This lesser increase in cellular Fe:C ([Fe:C]) occurred because at steady state, the [Fe:C] equals the iron uptake rate (V_Fe_) divided by the specific growth rate (μ):

(1) $$[\mathrm{Fe}:C]\;=\;V_{\mathrm{Fe}}/\mu$$

and the 15-fold increase in V_Fe_ is largely countered by a 5-fold increase in specific growth rate (**Figures 1A,C**).

Equation 1 dictates that the cellular Fe:C should increase as the growth rate is decreased by light intensity, provided the uptake rate is unaffected or decreases by less on a relative basis than the decrease in specific growth rate. This behavior is indeed observed: the curves for iron uptake rate vs. [Fe′] are similar at the three light intensities (**Figure 1C**); and at a given [Fe′], the Fe:C values in the slower-growing cells at the lowest light intensity are on average 49 ± 18% higher (± SD, *n* = 6) than values in the more rapidly growing cells at the highest light intensity within the [Fe′] range where the growth rates are not too severely iron-limited ([Fe′] ≥ 100 pM; **Figure 1B**). Similar increases in cellular Fe:C ratios with light limitation of growth rate have been observed in coastal eukaryotic phytoplankton species (Sunda and Huntsman, 1997, 2011). This increase provides some of the additional cellular iron needed for low light acclimation of photosynthesis.

The growth rate should increase with increasing cellular Fe:C in iron-limited cells as predicted from theory (Raven, 1990) and observations with eukaryotic algae (Sunda and Huntsman, 1995, 1997). This behavior is also observed in the present experiments, but only under mild to moderate iron-limitation of growth rate (μ ≥ 0.13 d^-1^; **Figure 3**). Here the cellular Fe:C ratio needed to support a given growth rate and the value needed to achieve μ_max_ increased with decreasing light intensity (**Figure 3**), as found previously for iron-light co-limitation of marine eukaryotic algae (Sunda and Huntsman, 1997). However, under severe iron-limitation of growth rate (μ = 0–0.08 d^-1^) cellular Fe:C ratios were the same or higher than those observed at higher, less iron-limited growth rates, indicating an uncoupling between iron growth rate limitation and cellular iron. The cellular Fe:C ratios showed the same pattern for the four sets of treatments: they first decreased by an average of 60 ± 4% (±SD, *n* = 4) as [Fe′] was decreased from the highest levels down to 48–100 pM (with the range in [Fe′] caused by the variation in light intensity). They then increased by 45 ± 17% with further decreases in [Fe′] from 48–100 to 16–36 pM (**Figure 1B**). With continued further decreases in [Fe′] within the lowest range (from 16–36 pM down to 4.7–9.4 pM), the cellular Fe:C values then decreased again by an average of 29 ± 12%. The interim increase in cellular Fe:C ratios with [Fe′] decreases from 48–100 to 16–36 pM was associated with the transition from mild to moderate iron-limitation of growth rate (reductions of 20–62%) to severe iron-limitation (growth rate reductions of 85–100%).

The efficiency of the metabolic iron use for C-fixation and growth [the iron use efficiency (IUE) in units of net moles of carbon [C] fixed per mol of intracellular Fe per day] can be computed by dividing the specific growth rate by the cellular Fe:C ratio (Raven, 1990). As the growth rate increases with increasing cellular Fe:C, the IUE increases to a maximum at a growth rate just below the maximum iron-sufficient rate at a given light intensity and then decreases with further increases in cellular Fe:C (**Figure 4**). Maximum iron use efficiencies (IUE_max_) in *S. bacillaris* decreased by 2.7-fold (from 4.9 to 1.8 kmol C [mol Fe]^-1^ d^-1^) with decreases in light intensity (**Table 1**), reflecting the increasing under-saturated state of the PA with respect to light absorption (Raven, 1990; Sunda and Huntsman, 1997). At any given light intensity, the IUE_max_ values were 4.7- to 9.4-fold lower than previously observed in coastal eukaryotic phytoplankton species under the same light conditions, indicating a much lower metabolic Fe use efficiency in the coastal cyanobacterial species (**Table 1**).

**Table 1:** Maximum iron use efficiencies (IUE_max_) and maximum iron-sufficient growth rates (μ_max_) for *Synechococcus bacillaris* and two coastal eukaryotic species: the diatom *Thalassiosira pseudonana* (Sunda and Huntsman, 2011) and the dinoflagellate *Prorocentrum minimum* (Sunda and Huntsman, 1997).

Species	Light intensity (μmol quanta m^-2^ s^-1^)	Maximum IUE (kmol C [mol Fe]^-1^ d^-1^)	μmax (d^-1^)
*Synechococcus bacillaris*	500	4.86±0.48	0.617±0.007
	160	3.9	0.59
	50	1.77	0.34
*Thalassiosira pseudonana*	500	45.9±1.9	1.78±0.09
	50	8.9	0.48
*Prorocentrum miminum*	500	35.2	0.61
	50	8.4	0.32

Iron-limitation of growth rate was accompanied by a decrease in cellular Chl *a*:C ratios (**Figure 5**), reflecting a decrease in photosynthetic units as the iron needed to synthesize critical iron proteins in these units decreased (Sunda and Huntsman, 1997, 2011). The Chl *a*:C ratio associated with a given growth rate was ∼2-fold higher at a light intensity of 160 μmol photons m^-2^ s^-1^, and ∼4-fold higher at an intensity of 50 μmol photons m^-2^ s^-1^ than that observed at the highest light intensity (**Figure 5**). The decrease in Chl *a*:C ratios with iron-limitation of specific growth rate occurred over the full range of growth rates, similar to the behavior observed previously in iron-limitation experiments with eukaryotic phytoplankton species (Sunda and Huntsman, 1995, 1997, 2011).

## Discussion

### Iron-Light Co-limitation of Growth Rate

The relationships between specific growth rate and cellular Fe:C at the varying light intensities were similar to those seen previously in coastal eukaryotic phytoplankton (Sunda and Huntsman, 1997, 2011) with some notable exceptions. As with eukaryotic algae, higher cellular Fe:C ratios were needed to achieve a given growth rate under low-light conditions for mild to moderate iron-limitation (**Figure 3**). This increase is caused by an up-regulation of the PA at low light intensity and an associated increase in the iron-containing cytochromes and FeS proteins within the PA (Raven, 1990; Strzepek and Harrison, 2004). As Chl *a* and other light harvesting pigments become under-saturated with respect to light absorption under low light intensity, a larger number of iron-containing photosynthetic units (consisting of light harvesting pigments, photosynthetic reaction centers, and electron transport proteins) are required to achieve a given rate of photosynthetic C-fixation and growth (Raven, 1990; Sunda and Huntsman, 1997). Between 50 and 90% of the cellular iron is contained in the PA based on model calculations (Raven, 1990) and empirical measurements (Strzepek and Harrison, 2004), so a substantial increase in photosynthetic units under low light acclimation means a large increase in both Chl *a* and cellular iron as we observed (**Figures 3** and **5**).

### Evidence for Cellular Accumulation of Non-Metabolic Iron under Severe Iron-Limitation

Positive correlations among cellular Fe:C ratios, Chl *a* and specific growth rate have been observed previously in iron-limited eukaryotic phytoplankton (Sunda and Huntsman, 1995, 1997, 2011), as expected from metabolic models (Raven, 1990). However, such behavior was observed only under mild to moderate iron-limitation (μ ≥ 0.13 d^-1^) in the present experiments. In cells growing at severely iron-limited rates (≤0.08 d^-1^) there was an uncoupling between specific growth rate and Chl *a* on the one hand and cellular Fe:C ratios on the other, in which the Fe:C ratios were unusually high relative to those projected to be needed to support the extremely low specific rates of C-fixation and growth (**Figure 3**) or those expected to be associated with the low cellular Chl *a*:C ratios (**Figure 5**). This behavior suggests that under severe iron-limitation, much of the measured intracellular iron is not associated with critical iron-proteins needed to support photosynthesis and growth. Rather it may be “stuck” in some intermediary cellular pool(s) that cannot be readily accessed for the synthesis of functional iron-proteins.

To examine this hypothesis, we need to look at the processes involved in cellular iron uptake and assimilation. Early reports suggested that marine *Synechococcus* species utilized siderophores (high affinity ferric chelators released into the external medium) for high-affinity iron uptake (Wilhelm and Trick, 1994). However, more recent genomic and meta-genomic data show little evidence for siderophore-mediated transport systems in most strains of *Synechococcus* and other picocyanobacteria (Hopkinson and Morel, 2009; Hopkinson and Barbeau, 2012). Instead marine cyanobacteria appear to largely utilize Fe(III) ABC transport systems to assimilate ferric iron (Hopkinson and Barbeau, 2012; Morrissey and Bowler, 2012). Iron uptake by these systems is thought to involve several steps: (1) diffusion of small ferric iron complex species (e.g., Fe′) through pores (porins) in the outer cell membrane, (2) ligand-exchange of these iron species with an iron-binding protein (often an IdiA homolog) within the periplasm (the space between the outer membrane and inner cytoplasmic membrane), and (3) ATP-dependent uptake of iron into the cytoplasm by a transport protein embedded in the cytoplasmic membrane (Webb et al., 2001; Morrissey and Bowler, 2012). The IdiA protein (and likely also the cytoplasmic membrane Fe-transporter) is substantially up-regulated under low-iron stress (Webb et al., 2001). The iron-IdiA protein complex itself does not appear to be transported into the cytoplasm, indicating that iron bound to the IdiA homolog is exchanged with a receptor site on the cytoplasmic membrane ferric iron transport protein prior to iron uptake into the cytoplasm by an as yet unidentified mechanism (Webb et al., 2001). Alternatively, ferric iron can be taken up via the reduction of ferric species [Fe′, the Fe(III)-IdiA protein complex, or other ferric complexes] to Fe(II) within the periplasm by energy (NADPH) driven transmembrane reductases and subsequent uptake of Fe(II) into the cytoplasm by a plasma membrane Fe(II) transport protein (e.g., FeoA/B; Morrissey and Bowler, 2012; Kranzler et al., 2014). Once inside the cell the iron is inserted into critical proteins needed for photosynthesis and growth by processes that may involve reducing agents (e.g., reduced glutathione) to reduce Fe(III) to much more labile Fe(II) to facilitate ligand exchange. At extremely low rates of photosynthesis in severely iron-limited cells, and associated low photosynthetic production of ATP and reducing equivalents (Raven, 1990), the cells may be unable energetically to transport the iron bound to the IdiA homolog into the cell or to insert intracellular iron into critical iron proteins. However, because the binding of ferric ions with the IdiA homolog likely occurs by simple ligand exchange reactions with labile ferric iron species (e.g., Fe′), this process could continue in slow-growing (or non-growing) cells under extreme iron/light limitation, resulting in an accumulation of ferric-IdiA protein complexes within the periplasm. This sequestered iron could then become available for cellular uptake and assimilation once the cell’s energetics are reestablished. Such a mechanism might provide a means for metabolically inactive cells under severe iron- or light-limitation to accumulate the iron needed to support future metabolism and growth. And it could provide another biological advantage at low iron availability by sequestering iron and thereby keeping it away from competing phytoplankton.

Evidence for unusually high accumulation of iron under severe iron-limitation of growth rate has been observed previously in six coastal and oceanic strains of *Synechococcus*, including our experimental strain *S. bacillaris* (CCMP 1333; Brand, 1991). In this study, cellular Fe:P molar ratios were estimated in stationary phase (non-growing) cultures from relationships between iron and phosphorus (P) limitation of cell yields in batch cultures. The estimated mean cellular Fe:P molar ratio in these experiments was 0.022 ± 0.013 (±SD), with the ratio for *S. bacillaris* (0.025) close to the mean value. Based on the C:P molar ratio in *Synechococcus* (132 ± 21; Bertilsson et al., 2003), the mean cellular Fe:C ratio in these stationary phase cultures would be 167 μmol mol^-1^ and the F:C ratio for *S. bacillaris* would be 189 μmol mol^-1^, close to the values we observed (88–185 μmol mol^-1^) in severely iron-limited cultures of *S. bacillaris* (**Figure 3**). However, as shown in our present experiments, much of this cellular iron was likely non-metabolic.

Unusually high Fe:C in severely iron-limited cells was also observed by Quesnel (2009). She found that an iron-limited oceanic *Synechococcus* strain (CCMP 3370) growing at low specific growth rate (0.20 d^-1^) had a cellular Fe:C ratio (117 ± 17 μmol mol^-1^; ±SD) that was higher than the value (109 ± 14 μmol mol^-1^) of iron sufficient cells growing at their maximum rate (0.42 d^-1^). The iron-limited value is very close to the average cellular Fe:C ratio (119 ± 27 μmol mol^-1^) measured in the eight severely iron-limited *S. bacillaris* cultures in the present study.

### Comparison of Cellular Iron Uptake Rates in *S. bacillaris* and Eukaryotic Algae

The current data provide an opportunity to compare the iron uptake systems in a marine cyanobacterial species with those of eukaryotic phytoplankton. Recent genomic and molecular data and experimental culture data indicate that ferric iron is taken up by eukaryotic algal species such as green algae (*Chlamydomonas reinhardtii*) and diatoms (e.g., *Thalassiosira oceanica*; *T. pseudonana, and T. weissflogii*) by the same system as is found in yeast (*Saccharomyces cerevisiae*), but one quite different from that used by cyanobacteria (Maldonado and Price, 2001; La Fontaine et al., 2002; Shaked et al., 2005). In the eukaryotic uptake system, soluble ferric hydrolysis species (Fe′) and ferric complexes are first reduced by transmembrane reductases at the cell surface to much more labile Fe(II), which greatly increases ligand-exchange kinetics and associated iron uptake rates (Maldonado and Price, 2001; Shaked et al., 2005). The released Fe(II) then binds to a membrane transport protein via ligand exchange reactions and is taken up into the cell in a process that involves re-oxidation of Fe(II) to Fe(III) by a multi-copper oxidase (La Fontaine et al., 2002). Relationships between cellular Fe uptake rates per unit of cell surface area and [Fe′] were the same in iron-limited coastal diatoms and dinoflagellates representing a range of cell diameters (3.5–32 μm), suggesting that they all were utilizing the same or similar iron transport systems (Sunda and Huntsman, 1997). The results indicated that larger cells were disadvantaged with regard to iron uptake because of their low surface to volume ratios.

**FIGURE 6:**
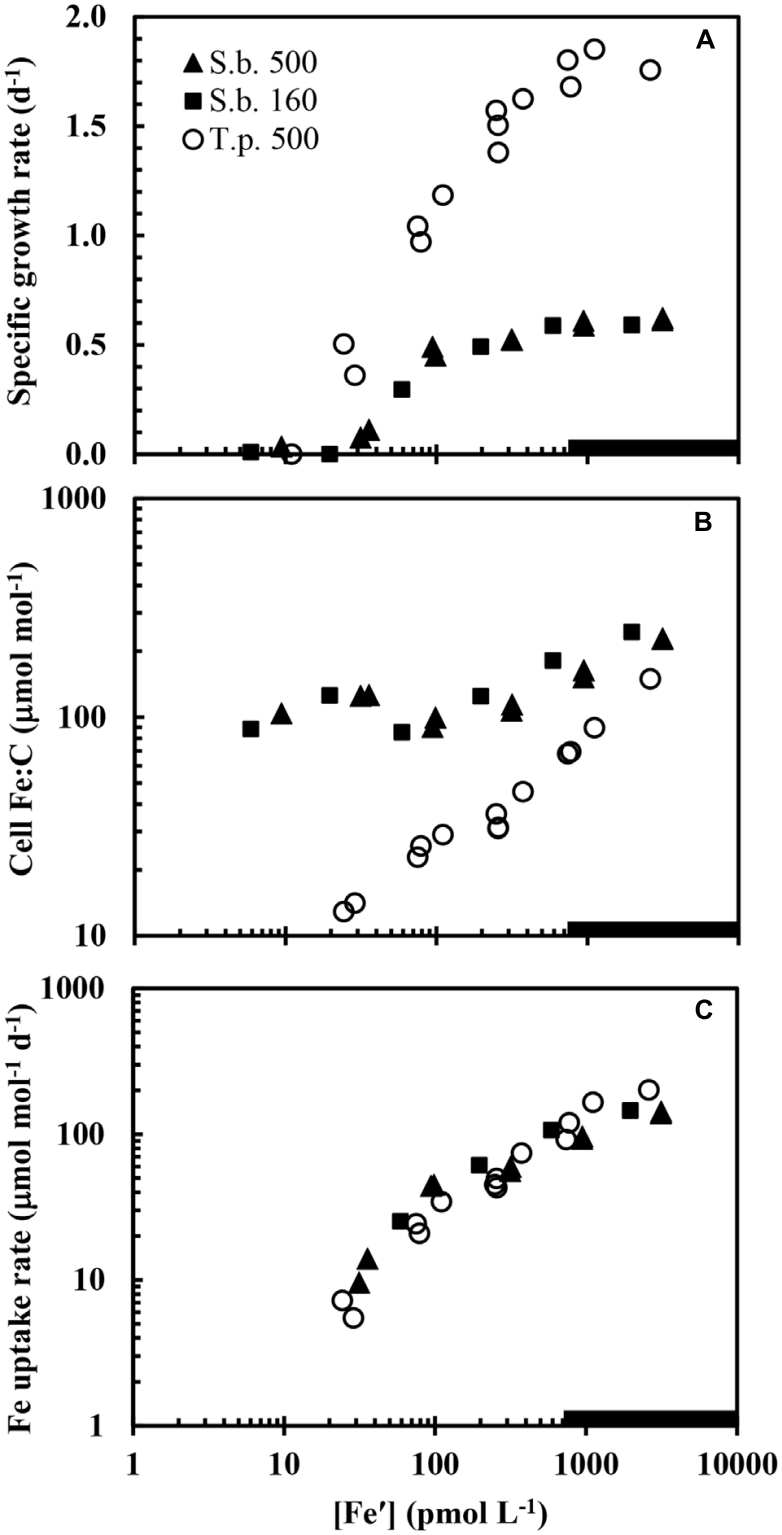
**Comparison of **(A)** specific growth rate, **(B)** cellular Fe:C, and **(C)** the cellular iron uptake rate normalized to cell carbon in *S. bacillaris* in the present study at the two higher light intensities (500 and 160 μmol quanta m^-2^ s^-1^; solid triangles and squares, respectively) and in the coastal diatom *T. pseudonana* in previous experiments (Sunda and Huntsman, 2011) measured at high light intensity (500 μmol quanta m^-2^ s^-1^; open circles) and the same temperature and photoperiod.** Data for *S. bacillaris* at high light (500 μmol quanta m^-2^ s^-1^; solid triangles) are for both low Cu and high Cu cultures ([Cu^2+^] = 0.16 and 16 pM, respectively).

Iron uptake rates in *S. bacillaris* normalized to cell carbon were compared to those of the diatom *T. pseudonana*, one of the four coastal eukaryotic phytoplankton species examined by Sunda and Huntsman (1997) in the same culture medium, at the same temperature, and under the same lighting system as used in our present experiments. The relationships between iron uptake rate per mol of cell C and [Fe′] were very similar in the prokaryotic and eukaryotic species (**Figure 6C**). However, this similarity is misleading because of the ∼2.6-fold larger cell diameter in *T. pseudonana* (3.5–4.4 μm; Sunda and Huntsman, 1995) than in *S. bacillaris* (∼1.5 μm; https://ncma.bigelow.org), and the resultant equivalent decrease in the cell surface to volume ratio. Thus, given the similarity in uptake rates per unit of cell C (**Figure 6C**) and the larger surface to volume ratio in the smaller cyanobacterial species, the uptake rate normalized to surface area should be higher in *T. pseudonana* than in *S. bacillaris* by ∼2- to 3-fold, suggesting that the coastal diatom has a more efficient iron uptake system, at least under our experimental conditions. This result is not unique to *T. pseudonana* as in similar experiments an oceanic species of this genus (*T. oceanica*, cell diameter 5–6 μm) had iron uptake rates (for a given [Fe′]) that were also similar to those for *S. bacillaris*, while the iron uptake rates for an oceanic pelagophyte (*Pelagococcus calceolatta*; ∼2 μm diameter) and a widely distributed oceanic prymnesiophyte (*Emiliania huxleyi*; 3 μm diameter) were 2- to 3-fold higher than those for *S. bacillaris* at low Fe′ concentrations (Sunda and Huntsman, 1995). The high-affinity iron uptake system in *T. pseudonana, T. oceanica* and other eukaryotic phytoplankton is dependent on copper (La Fontaine et al., 2002; Maldonado et al., 2006). The lack of an appreciable copper effect on iron uptake and growth rate in the present experiments (**Figures 1A,C**) is consistent with there being a different high-affinity iron uptake system in *S. bacillaris* as discussed above.

### Higher Cellular Iron Requirements for Growth in *Synechococcus* than in Eukaryotic Algae: A Vestige of Early Evolution?

We observed unusually high cellular iron requirements for growth (**Figure 3**) and associated low maximum iron use efficiencies in *S. bacillaris* (**Table 1**) relative to values observed in coastal eukaryotic phytoplankton. At the highest light intensity, *S. bacillaris* required a cellular Fe:C ratio of 111 ± 5 (±SD) μmol mol^-1^ to grow at an iron-limited rate of 0.52 d^-1^, while coastal eukaryotic species (the diatoms *T. pseudonana* and *T. weissflogii* and the dinoflagellate *Prorocentrum minimum*) needed cellular Fe:C ratios of only ∼13–16 μmol mol^-1^ (12–14% as much) to grow at the same iron-limited specific rate under the same temperature and light conditions (Sunda and Huntsman, 1997, 2011).

One reason for the much higher iron requirements for growth in *S. bacillaris* is likely the difference in the stoichiometry of the PA, which as noted earlier, contains 50–90% of the cellular iron quota, with the higher percentages occurring in low-light acclimated cells (Raven, 1990; Strzepek and Harrison, 2004). Photosynthesis involves linear electron flow between five iron-containing proteins and protein complexes: photosystem II (PSII, which contains 2–3 Fe atoms), the cytochrome *b*_6_/*f* complex (5 Fe), soluble cytochrome *c*_6_ (1 Fe), photosystem I (PSI, 12 Fe) and ferredoxin (2 Fe; Raven, 1990; Strzepek and Harrison, 2004). This photochemically driven electron flow produces both energy [adenosine triphosphate (ATP)] and reducing equivalents [reduced ferredoxin and reduced nicotine adenine dinucleotide phosphate (NADPH)], which are needed for C-fixation and the reduction of nitrate to ammonium (Raven, 1990). In addition there can be cyclic flow of electrons around the lower end of the electron transport chain (the *b*_6_/*f* complex, Cyt *c*_6_, PSI, and ferredoxin), which produces ATP but no NADPH (Falkowski and Raven, 2007). One might expect there to be roughly equal amounts of each electron transfer component for smooth linear electron flow, but this is usually not the case. Instead the components of the transport chain can differ considerably from a balanced stoichiometry by allowing different electron carriers to cycle at different rates (Raven, 1990; Greene et al., 1991; Strzepek and Harrison, 2004). In a marine *Synechococcus* strain the ratio of the iron-inexpensive PSII (2–3 Fe) to iron-rich PSI (12 Fe) in iron-replete cells was 0.5 (Melis, 1989). By contrast the PSII to PSI ratios are 2.5–4.4 (5- to 9-fold higher) in iron-limited and iron-sufficient cultures of the coastal diatoms *T. weissflogii* (Strzepek and Harrison, 2004) and *Phaeodactylum tricornutum* (Greene et al., 1991) grown under saturating light. PSII to PSI ratios are even higher (9–11) in a diatom (*T. oceanica*) isolated from iron-poor oceanic waters (Strzepek and Harrison, 2004). In this and many other oceanic eukaryotic species, the Fe:C ratios needed to support a given growth rate are as little as 3–6% of that needed to support an equivalent growth rate in *S. bacillaris* (Sunda and Huntsman, 1995; Marchetti et al., 2006). Thus, eukaryotic species (especially oceanic ones) have been able to substantially lower their iron requirement for growth by decreasing the ratios of iron-expensive PSI to iron-inexpensive PSII, although the means by which they have done so and still maintain viable rates of photosynthesis are not known.

Since oceanic eukaryotic species have lower cellular iron requirements for growth than coastal ones (Sunda and Huntsman, 1995; Maldonado and Price, 1996; Marchetti et al., 2006) due to adaptations to the much lower iron concentrations in oceanic realms than in coastal waters, one might expect that evolutionary pressures would also have led to a similar decrease in cellular growth requirements for iron in oceanic *Synechoccocus* species and strains. However, the limited amount of iron data for oceanic and coastal *Synechococcus* strains largely does not support this hypothesis. In a study of two coastal *Synechococcus* strains (PCC7002 and WH5701) and two oceanic ones (WH8102 and WH7803), the oceanic strains were more sensitive to iron-limitation than the coastal ones (Liu and Qiu, 2012), the opposite of what is found with eukaryotic phytoplankton. Likewise, two coastal strains (CCMP 2515 and 838) required cellular Fe:C values of 32.3 and 38.6 μmol mol^-1^ to grow at an iron-limited specific rate of 0.4 d^-1^, while three oceanic strains (CCMP 837, 1134, and 2373) required higher cellular Fe:C ratios (44.1, 83.4, 117 μmol mol^-1^, respectively) to grow at the same or lower iron-limited rates (0.2–0.4 d^-1^; Quesnel, 2009). These Fe:C values are similar to that (82 μmol mol^-1^) for *S. bacillaris* growing at a similar iron-limited rate of 0.3 d^-1^ in the present experiments at a light intensity of 160 μmol quanta m^-2^ s^-1^. Making such comparisons, however, are not entirely straight-forward as cellular iron requirements for growth increase not only with decreasing light intensity, but also with decreasing photoperiod and temperature (Sunda and Huntsman, 2011), and all three of these parameters were different in the two sets of experiments: those of Quesnel (2009) were conducted at 23°C under continuous light at a light intensity of 50 μmol quanta m^-2^ s^-1^ while the Fe:C value noted above in our experiments was for cells grown at a lower temperature (20°C), shorter photoperiod (14 h d^-1^), and higher light intensity (160 μmol quanta m^-2^ s^-1^). The above studies suggest that oceanic *Synechococcus* strains have the same or slightly higher iron growth requirements with regard to both external iron (Fe′ concentration) and cellular Fe:C ratios. Likewise in a study of six strains of *Synechococcus*, Brand (1991) found no consistent differences in the subsistence Fe:P ratios between coastal and oceanic isolates.

The cellular iron growth requirements for oceanic and coastal *Synechococcus* isolates discussed above are higher than those needed to support the growth of eukaryotic coastal algae (10–20 μmol mol^-1^ to support iron-limited specific growth rates of 0.3–0.7 d^-1^ under 14 d^-1^ of saturating light at 20°C) and much higher than those needed to support the growth of oceanic eukaryotic algae at iron-limited specific growth rates of 0.7–0.9 d^-1^ (3–5 μmol mol^-1^) under the same growth conditions (Sunda and Huntsman, 1995, 1997, 2011). So both coastal and oceanic *Synechococcus* appear to have much higher iron requirements for growth than oceanic eukaryotic algae, a pattern also born out by comparisons of subsistence Fe:P ratios between six strains of coastal and oceanic *Synechococcus* and an equivalent number of oceanic eukaryotic algal species (Brand, 1991). Likewise, in another study, the growth of the oceanic *Synechococcus* strain WH7803 (aka DC2) was much more iron-limited than that of all 11 oceanic eukaryotic algal species examined in the same experiments (Brand et al., 1983).

The much higher cellular requirement for iron in *S. bacillaris* and other *Synechococcus* strains than in coastal and oceanic eukaryotic species may represent a vestige of early evolution when iron availability was much higher than it is today, and there was much less need to economize with respect to the metabolic use of iron. This idea was previously proposed by Brand (1991) and Saito et al. (2003), and our current data provide support for this hypothesis. As noted earlier, O_2_-producing cyanobacteria evolved ca. 3 billion years ago when the oceans were reducing, and contained high concentrations of iron in the form of much more soluble Fe(II) (Blankenship et al., 2007). The photosynthetically generated O_2_ gradually accumulated over 100s of millions of years, first in the atmosphere and surface ocean between 2.5 and 0.5 billion years ago, and then in the ocean as a whole (except for a few anoxic basins) beginning around 500 million years before present (Anbar and Knoll, 2002). This gradual titration of the ocean with O_2_ resulted in the oxidation of Fe(II) to Fe(III), and subsequent precipitation of insoluble Fe(III) oxides, which ultimately led to the low oceanic iron concentrations we see today. The main eukaryotic phytoplankton groups that currently dominate the productivity of the ocean (diatoms, dinoflagellates, and prymnesiophytes) first appeared in the ocean during the late Triassic to early Jurassic Periods (ca. 200 million years ago; Katz et al., 2007) when the oceans were oxygenated, and iron would have existed at low concentrations as sparingly soluble Fe(III) (Blankenship et al., 2007). Thus, over time, marine algae have had to adapt to an ever decreasing availability of iron by altering the stoichiometry of the PA (Strzepek and Harrison, 2004), and replacing iron-containing photosynthetic redox proteins with those that contain no iron (replacing ferredoxin with flavodoxin; La Roche et al., 1996) or contain another more available metal redox center (replacing cytochrome c with the copper protein plastocyanin Peers and Price, 2006). The evolution of a highly effective copper-dependent iron uptake system in eukaryotic algae (La Fontaine et al., 2002; Maldonado et al., 2006) also should have contributed to this adaptation.

*Synechococcus bacillaris* and other cyanobacterial species and strains possess several other unusual trace metal requirements and sensitivities which may also be adaptive vestiges of an earlier anoxic and sulfidic ocean. Due to the high sulfide concentrations in the ancient anoxic ocean, trace metals which form highly insoluble sulfides (zinc, copper, and cadmium) would have been present at exceedingly low concentrations, while others whose sulfides are much more soluble (cobalt [Co], nickel [Ni], iron [Fe], and manganese) would have been more available for incorporation into metabolic systems (da Silva and Williams, 1991; Saito et al., 2003). With the advent of photosynthetically released oxygen, the insoluble sulfides were oxidized to sulfate, thereby releasing zinc, copper, and cadmium into ocean waters, while it oxidized soluble Co(II) to insoluble Co(III) oxides and Fe(II) to sparingly soluble Fe(III), thereby substantially decreasing Co and Fe concentrations. Thus, the modern ocean is believed to be much richer in zinc, copper, and cadmium, but poorer in iron and cobalt. Brand et al. (1983) found that *S. bacillaris* and other *Synechococcus* strains were much more sensitive to copper and cadmium toxicity than eukaryotic algal species and suggested that this might be a vestige of the much lower copper and cadmium concentrations in the ancient reducing ocean. Likewise, Sunda and Huntsman (1995) observed that *S. bacillaris* had an absolute requirement for cobalt, but not for zinc, in contrast to the situation with eukaryotic phytoplankton (e.g., diatoms), which have an absolute requirement zinc, which can partly or wholly be replaced by cobalt. A similar absolute requirement for cobalt but not zinc was observed in a strain of the oceanic cyanobacterium *Prochlorococcus* (Saito et al., 2002). Both sets of authors suggested that these unusual cobalt requirements might be a vestige of the higher cobalt and lower zinc availability in the ancient reducing ocean. In addition many marine *Synechococcus* and other cyanobacteria (especially oceanic species and strains, e.g., of *Prochlorococcus*) contain Ni-dependent superoxide dismutases and other Ni-dependent enzymes (e.g., urease, and hydrogenases), while with the exception of urease, these Ni-enzymes are absent in marine eukaryotic algae (Dupont et al., 2008; Qui and Price, 2009). Thus not only the unusually high requirements for iron in marine cyanobacteria (e.g., *Synechococcus*), but also their unique metabolic needs for cobalt and nickel, the absence of an absolute zinc growth requirement, and high sensitivities to the toxic metals copper and cadmium may be vestiges of the much different trace metal conditions in the early ocean when these organisms first evolved (Brand et al., 1986; Sunda and Huntsman, 1995; Saito et al., 2002).

Because *S. bacillaris* has such a high cellular iron requirement for growth, one might expect its growth rate to be limited at a much higher Fe′ concentration than the growth of coastal eukaryotic species. However, curves for μ/μ_max_ (the iron-limited rate divided by the maximum iron sufficient growth rate) vs. [Fe′] for *S. bacillaris* at our three experimental light intensities are independent of light intensity and virtually identical to those for the coastal diatom *T. pseudonana* and dinoflagellate *P. micans* (**Figure 2**; Sunda and Huntsman, 1997). Growth limitation in all of these coastal species occurred at Fe′ concentrations just below the solubility limit for Fe′ with respect to precipitation of ferric hydroxides, irrespective of light intensity (**Figure 2**). It has been argued that such similarity in iron requirements among coastal species is no mere coincidence (Sunda and Huntsman, 1997). Rather, it was argued that the precipitation of ferric hydroxides sets an upper limit on iron solubility and Fe′ concentrations in seawater, and therefore, on the availability of iron to support photosynthesis and growth in phytoplankton, especially in coastal seawater where the supply of iron from continental sources is high (Sunda and Huntsman, 1997). Thus, the ferric iron solubility limit may have restricted the maximum growth rate of coastal phytoplankton species, including *S. bacillaris*, under varying light conditions.

### Biological Consequences of a High Iron Growth Requirement

The high cellular iron requirement for growth may have also restricted the size of *S. bacillaris* and other *Synechococcus* species and strains. Marine *Synechococcus* cells have diameters of 0.7–1.5 μm and are among the smallest of the marine phytoplankton (Waterbury et al., 1979). Their small size and resultant high surface to volume ratios allow them to have biomass-normalized iron uptake rates equal to or greater than much larger eukaryotic algal species, despite an apparent lower iron uptake rate per unit of cell surface area as discussed above (see **Figure 6C**). However, their high iron requirement for photosynthesis and growth also restricts the growth of these cells to low specific rates. This low growth rate has two important effects. First, as predicted from Eq. 1, it allows *Synechococcus* to maintain higher Fe:C ratios than faster growing phytoplankton cells due to a lower biodilution rate. For example, the lower maximum growth rate of *S. bacillaris* (0.6 d^-1^ at 20°C) allows it to maintain much higher steady-state cellular Fe:C ratios than the faster growing coastal diatom *T. pseudonana* (μ_max_ = 1.8 d^-1^ at the same temperature; **Figures 6A,B**) despite the similarity in carbon-normalized iron uptake rates (**Figure 6C**). And a lower maximum growth rate lessens the amount of cellular iron needed to support that lower rate.

The high cellular iron requirement for growth in *S. bacillaris* and other *Synechococcus* species or strains suggests that the availability of iron relative to that of major nutrients (N and P) is important in regulating the distribution of these cyanobacteria in the ocean. *Synechococcus* is widely distributed in coastal waters and in near-surface, thermally stratified oceanic waters (Olson et al., 1990; Flombaum et al., 2013), where N or P, but not iron, generally limits phytoplankton growth rates (Ryther and Dunstan, 1971; Sanders et al., 1987; Moore et al., 2013). However, thermally stratified ocean waters usually possess a DCM at the bottom of the photic zone arising both from low light acclimation of the phytoplankton community and resultant increases in cellular Chl *a*:C ratios, and also to local maxima in primary production and phytoplankton biomass linked to diffusive flux of new nitrogen (i.e., nitrate) and other nutrients (Fe and P) from deeper aphotic depths (Cullen, 1982; Wawrik et al., 2003; Huisman et al., 2006; Hopkinson and Barbeau, 2008). Unlike phytoplankton growth nearer the surface which is limited by N or co-limited by N and P (Moore et al., 2013), that in the DCM was thought to be limited by light or co-limited by light and nitrogen (Cullen, 1982; Huisman et al., 2006). Based on the linkages between light and iron-limitation in eukaryotic marine algae and by the presence of a pronounced minimum in filterable iron concentrations at the depth of the DCM (Bruland et al., 1994), Sunda and Huntsman (1997) proposed that phytoplankton growth in the DCM was co-limited by iron and light. This hypothesis was later supported by at-sea iron and light manipulation experiments (Hopkinson and Barbeau, 2008) and by the widespread occurrence of iron minima at the depth of DCM in the central gyres of the North Atlantic and Pacific (Bruland et al., 1994; Boyle et al., 2005; Sedwick et al., 2005). In fact phytoplankton growth in the DCM may be co-limited by light, iron, and nitrogen given the high iron requirement for the reductive assimilation of nitrate (Raven, 1988; Maldonado and Price, 1996; Kudo and Harrison, 1997) fluxing in from aphotic depths.

Our current results also support iron-light co-limitation of algal growth and iron-light-nitrogen co-limitation of new (nitrate supported) phytoplankton production within the DCM. Our results and those of others indicate that *Synechococcus* has unusually high iron requirements for photosynthesis and growth, and despite the small size of *S. bacillaris* (∼1.5 μm diameter), its iron uptake rates normalized to cell carbon were similar to those for larger eukaryotic algae such as the coastal diatom *T. pseudonana* (∼4 μm diameter; **Figure 6C**), and the oceanic diatom *T. oceanica* (∼5 μm diameter; Sunda and Huntsman, 1995). Thus, *Synechococcus* should be at a growth disadvantage relative to eukaryotic phytoplankton in the low-light, low-iron waters of the DCM. In support of this prediction, *Synechococcus* is observed to decrease as a percent of phytoplankton biomass with depth in the DCM with decreases in light, and likely also iron based on detailed depth profiles of iron concentrations (Bruland et al., 1994; Boyle et al., 2005; Sedwick et al., 2005). By contrast the biomass at the depth of the DCM is often dominated by small eukaryotic algae (picoeukaryotes) such as pelagophytes and prymnesiophytes (Li et al., 1992; Campbell and Vaulot, 1993; McManus and Dawson, 1994; Li, 1995; Wawrik et al., 2003), whose Fe′ and cellular Fe:C requirements are much less than found in *S. bacillaris* and other strains of *Synechococcus* (Brand et al., 1983; Sunda and Huntsman, 1995; Kudo and Harrison, 1997; Quesnel, 2009). These data suggest that the high iron requirement for growth and low light acclimation in *Synechococcus* largely excludes this genus from the low-iron, low-light waters of the DCM. Thus, we propose that iron-light co-limitation helps structure the phytoplankton community within the DCM.

The depth distribution of another cyanobacterial group, *Prochlorococcus*, also supports this hypothesis. This genus is comprised of two major genetically distinct ecotypes: a high light ecotype that is widely distributed within the light-sufficient, but major nutrient-limited surface mixed layer of the stratified tropical and subtropical ocean, and a low-light ecotype that is orders of magnitude less abundant within the upper photic zone but is by far the dominant type within the light-limited DCM (Rocap et al., 2003; Biller et al., 2015). In experiments conducted in Fe-EDTA buffered seawater media, a low-light strain (MIT9313), isolated from within the DCM (135 m depth) of the subtropical northwestern Atlantic, was an order of magnitude less sensitive to iron-limitation than a high-light strain (MED4), isolated from a depth of 5 m from the Mediterranean Sea (Thompson et al., 2011). The growth rate of the low-light strain was reduced to zero at Fe′ concentrations ≤1 pM, whereas that of the high-light strain was reduced to zero at 20-fold higher Fe′ levels (≤20 pM). These results are consistent with the low-light ecotype being adapted to growth at the low iron concentrations and low light levels of the oceanic DCM. However, based on the data for growth rate vs. Fe′ concentration, this ecotype is still more sensitive to iron-limitation than oceanic eukaryotic algae such as prymnesiophytes and pelagophytes (Sunda and Huntsman, 1995), which typically dominate the biomass of the mid to lower DCM (Li et al., 1992; Campbell and Vaulot, 1993). Thus, there is a distinct layering of algal communities in stratified ocean waters, with *Synechococcus* and the high-light *Prochlorococcus* ecotype exhibiting high abundance in the well-lit surface layer and then declining rapidly with depth in the upper DCM; the low light *Prochlorococcus* ecotype showing an intermediate depth distribution, with maximum numbers in the upper DCM; and picoeukaryotes being abundant at all depths, but dominating the algal biomass in the mid to lower DCM (Li et al., 1992; Campbell and Vaulot, 1993; McManus and Dawson, 1994; Wawrik et al., 2003; Biller et al., 2015). This depth distribution matches the order of sensitivities of these phytoplankton groups to iron-limitation based on existing data. It is interesting to note that the high light ecotype of *Prochlorococcus* (MED4) had a similar high sensitivity to iron-limitation as *S. bacillaris*, with the growth rate of both species decreasing to zero at the same Fe′ concentration ([Fe′] ≤ 20 pM) in Fe-EDTA buffered culture media (**Figure 2**; Thompson et al., 2011). And both cyanobacterial groups show a sharp decline in abundance with decreasing light and iron concentrations with depth in the DCM, suggesting that iron-light co-limitation may be an important factor in these depth dependent declines.

If these speculations are correct, then iron is not only important in controlling the growth and species diversity of phytoplankton in the so-called high-nitrate, low chlorophyll regions which comprise ca 30% of the ocean’s surface (Moore et al., 2013), but also in the DCM of the vast stratified regions of the tropical and subtropical ocean that occupy most of the remaining ∼70% of ocean waters, and which are projected to increase in extent with future global warming (Flombaum et al., 2013). And even in these stratified ocean regions, phytoplankton productivity in near-surface waters can be viewed as often being co-limited by nitrogen and iron, because of the high iron-requirement for N_2_-fixation by diazotrophic cyanobacteria, which along with denitrification, controls the oceanic supply of fixed N (Sohm et al., 2011; Sunda, 2012). Thus, it can be argued that iron is fundamentally the most limiting nutrient in the ocean, due both to its critical role in catalyzing key metabolic processes (photosynthesis, respiration, nitrate reduction, and N_2_-fixation), but also to an ever decreasing abundance of iron in ocean waters over geologic time linked to the evolution of oxygenic photosynthesis, which itself is highly dependent on iron.

## Conflict of Interest Statement

The authors declare that the research was conducted in the absence of any commercial or financial relationships that could be construed as a potential conflict of interest.
